# Putting benchmarks in their rightful place: The heart of computational biology

**DOI:** 10.1371/journal.pcbi.1006494

**Published:** 2018-11-08

**Authors:** Bjoern Peters, Steven E. Brenner, Edwin Wang, Donna Slonim, Maricel G. Kann

**Affiliations:** 1 La Jolla Institute for Allergy and Immunology, La Jolla, California, United States of America; 2 Department of Plant and Microbial Biology, University of California, Berkeley, California, United States of America; 3 Cumming School of Medicine, University of Calgary, Calgary, Alberta, Canada; 4 Department of Computer Science and Genetics, Tufts University, Medford, Massachusetts, United States of America; 5 Department of Biological Sciences, University of Maryland, College Park, Maryland, United States of America; UNITED STATES

## Abstract

Research in computational biology has given rise to a vast number of methods developed to solve scientific problems. For areas in which many approaches exist, researchers have a hard time deciding which tool to select to address a scientific challenge, as essentially all publications introducing a new method will claim better performance than all others. Not all of these claims can be correct. Equally, for this same reason, developers struggle to demonstrate convincingly that they created a new and superior algorithm or implementation. Moreover, the developer community often has difficulty discerning which new approaches constitute true scientific advances for the field. The obvious answer to this conundrum is to develop benchmarks—meaning standard points of reference that facilitate evaluating the performance of different tools—allowing both users and developers to compare multiple tools in an unbiased fashion.

Broadly speaking, benchmarks consist of input data that methods are meant to operate upon, expected output data against which tool output can be compared, a specification of metrics used to assess performance, and performance values of sets of tools that have been run through the benchmark.

Developing good and comprehensive benchmarks, in which the performance metrics of each tool reflect its real-world utility, requires a significant effort. For highly competitive and established fields, such as protein structure predictions, community experiments evaluating the methods have been held periodically to provide blinded assessments of prediction performance. These blinded assessments are perhaps the gold standard on how benchmarks should be run. However, in most areas of computational biology, no such regular blinded contests are available. Instead, many tool developers end up generating their own benchmarks, which they publish alongside a newly developed tool to show its improved performance. The downside of this approach is that, if a new approach is developed in parallel to assembly of the benchmark on which it is evaluated, there is a strong selection bias encouraging the authors to report tool development approaches performing well against the benchmark compared to previous tools. This reporting bias makes most benchmarks that accompany newly developed tools questionable. Even if the authors are aware of this problem and take conscious steps to separate benchmark, evaluation method, and method development, subconscious bias may persist and affect the final outcome.

One of the reasons why most journal articles that include benchmarks are accompanied by the introduction of new tools is that most prominent journals, including *PLOS Computational Biology*, prioritize articles that describe novel biological or methodological findings. A well-designed benchmark does neither. Benchmark-derived metrics should stand as proxies for tool utility to the user community, so benchmarks tied to specific tools are potentially suspect. Thus, there is a need to provide a prominent place to publicize benchmarks that are well designed and meet community needs.

To address this need, we are introducing a new article category in *PLOS Computational Biology* dedicated to benchmarks. Through a series of discussions within the editorial team, reviewers, authors, and other community members, we have assembled a tentative list of criteria that we ask reviewers to take under consideration when evaluating benchmark manuscripts (see Box 1). In this issue, we are publishing two articles that went through the regular review process as well as assessment for the following benchmark criteria. We expect that over time, the benchmark criteria will be refined in an iterative process as more manuscripts are reviewed.

We encourage community members working on manuscripts that fit these considerations to submit to this new section dedicated to benchmarking. Overall, we hope that creation of this section in *PLOS Computational Biology* will help elevate this research activity to where it belongs: the heart of computational biology.

**Box 1. Review criteria that can be considered (in no particular order): What makes a benchmark good?**

1. Methods being benchmarked must be in an active area of research.
2. Tools evaluated should be comprehensive, or at least judiciously selected, among those publicly available in the field.
3. The input- and expected output datasets used in the benchmark must be made freely available in a form that makes them easy to apply and reuse, to serve as validation datasets for new method development.
4. Benchmark results must be trustworthy. This criterion is best achieved by being completely transparent about how the benchmark was conducted and making the results reproducible, ideally by making code to perform the assessment publicly available.
5. Metrics used to measure tool performance should reflect different goals with practical relevance for potential users. There should be a limited number of metrics evaluating orthogonal aspects of tool performance (e.g., classification accuracy measured by an area under the curve (AUC) value and classification speed measured in seconds). If there are multiple distinct applications for the methods, inclusion of a suite of goal-specific datasets and metrics can be more appropriate.
6. Methods benchmarked should be publicly available. Tools that are available free of charge as executables or—even better—at the source code and platform level should be appropriately credited and given preference for inclusion in evaluations. The training conditions of each tool should be clearly indicated.
7. Novel tools created by the authors in parallel with the benchmark should not be included in the article, as they are essentially guaranteed to perform well. If they are included, this caveat should be prominently stated.

